# The relationship between polycyclic aromatic hydrocarbons exposure and serum klotho among adult population

**DOI:** 10.1186/s12877-022-02924-9

**Published:** 2022-03-14

**Authors:** Yuan-Yuei Chen, Wei-Liang Chen

**Affiliations:** 1 Department of Pathology, Tri-Service General Hospital, and School of Medicine, National Defense Medical Center, Taipei, Taiwan, Republic of China; 2 Department of Pathology, Tri-Service General Hospital Songshan Branch, and School of Medicine, National Defense Medical Center, Taipei, Taiwan, Republic of China; 3 Division of Geriatric Medicine, Department of Family and Community Medicine, Tri-Service General Hospital, and School of Medicine, National Defense Medical Center, Taipei, Taiwan, Republic of China; 4 Department of Biochemistry, National Defense Medical Center, Taipei, Taiwan, Republic of China

**Keywords:** Polycyclic Aromatic Hydrocarbons, Klotho, Adult

## Abstract

**Background:**

Klotho is an important factor involving aging process. Recently, polycyclic aromatic hydrocarbons (PAHs) exposure was reported to have adverse impact on DNA methylation associated with aging. The aim of the current study was to determine the relationship between serum klotho and PAHs exposure in an adult population.

**Methods:**

A total of 2597 participants obtained from NHANES 2015–2016 were included in this cross-sectional study. Serum klotho levels were analyzed by enzyme-linked immunosorbent assay (ELISA). PAHs exposure was estimated by urinary sample using liquid chromatography-tandem mass spectrometry. The relationship between serum klotho and exposure to PAHS was analyzed by a multivariable linear regression model.

**Results:**

2-napthol and 3-fluorene were significantly associated with decreased klotho. After fully adjusting pertinent variables, PAH exposure was significantly associated with decreased klotho, particularly in men.

**Conclusion:**

In the present study we highlighted the significant association between PAHs exposure and serum klotho levels. The importance of environmental effect on aging process and age-related disorders should be paid more attention and clinical intervention is necessary.

## Introduction

*Klotho* gene mutation was reported to involve many pathways about aging process such as shortened lifespan, vascular calcification, growth retardation, and osteoporosis [1–3]. It encodes a type I transmembranous protein related to β-glucuronidases and divides into α-, β-, and γ- isoforms [4, 5]. α-Klotho has emerged as a powerful regulator of the aging process by regulating many pathways, such as the regulation of phosphate homeostasis, Wnt signaling, and insulin signaling [5]. It is regarded as a crucial role in the pathophysiology of common aging related disorders, including chronic kidney disease, metabolic syndrome, and cardiovascular diseases [6–8].

Polycyclic aromatic hydrocarbons (PAHs) are organic compounds with multiple aromatic rings of only carbon and hydrogen [9]. These pollutants are generated via the insufficient combustion of natural or synthetic organic matter [10, 11], including biomass burning [12], vehicular exhausts [13], and metal emission [14]. They can spread from their source via many media such as air, water [15, 16], and even store in the terrestrial soils [13, 17]. Methods for evaluating PAHs exposure include job exposure matrices in exposure modeling, occupational settings, self-reported exposures, and biomarkers [18–20]. Hydroxylated (OH) PAH metabolites in urinary sample are widely used biomarkers to assess implied human exposure to a number of PAHs [21, 22]. Commonly detected urinary metabolites are low-molecular weight PAHs, including naphthalene, fluorene, phenanthrene, and pyrene [23]. Evidence reports that high-molecular weight PAHs with over 4 aromatic rings, especially benzo[a]pyrene, are mainly excreted via feces and are not detectable in urine [24–26].

PAH exposure causes various systemic diseases, including cancer, obstructive lung diseases, and cardiovascular diseases [27–29]. Recently, PAH exposure was suggested to be associated with frailty, including poor muscle strength and altered bone turnover in elder population [30, 31]. PAHs exposure may have adverse impact on aging process by accelerating DNA methylation aging [32]. To the best of knowledge, no previous study has reported the association between exposure to PAHs and klotho either in vivo or in vitro. In our study, we hypothesized that PAHs exposure may be correlated with decreased serum klotho in a cross-sectional analysis of a nationally representative general adult sample in the United States.

## Method

### Study population

All study information was collected from the National Health and Nutrition Examination Survey (NHANES) 2015–2016. NHANES is a major program of the National Centers for Health Statistics (NCHS). This nationally representative survey engages a variety of health and nutrition measurements of population in United States. A total of 2637 participants included in the NHANES database who had klotho in the serum measured from 2015 to 2016 were included in this study. After excluding those didn’t have laboratory data of urinary PAH and complete other examinations, 2597 eligible participants included 1318 men and 1279 women were enrolled in this study. Ethics approval was approved by the Institutional Review Board of the NCHS Ethics Review Board (number: Protocol #2011–17) and study design was confirmed in accordance with the Helsinki Declaration. Participants provided informed consent before enrollment.

### Level of urinary PAHs

The urinary hydroxylated polycyclic aromatic hydrocarbon metabolites included 1-naphthalene, 2-fluorene, 2-naphthalene, 1-phenanthrene, 3-fluorene, 3-phenanthrene, 2-phenanthrene, and 1-pyrene. Urinary sample was collected by a well-trained technician under standard protocol and were stored at -20 °C. The separation and quantification of urinary metabolites was using isotope dilution high performance liquid chromatography-tandem mass spectrometry [33]. To prepare for quality control materials, a known native standard mixture was digested into an anonymous filtered urine pool. The quality control solution was homogenized for at least 3 h then aliquoted into vials and stored at -70 °C [34]. A matrix matched standard calibration curve was used for comparison to target reagents, which was determined from comparative response of native to labelled standards in the urinary samples [33]. These samples were analyzed with one blank sample, one quality control (low), and another quality control (high level) simultaneously in each run. The limits of detection for PAHs metabolites ranged from 0.007 ng/mL to 0.09 ng/mL.

### Measurement of serum klotho

Analyses for serum klotho levels were performed by a commercially available enzyme-linked immunosorbent assay (ELISA) kit (Immuno-Biological Laboratories, Takasaki, Japan) according to the manufacturer’s protocol. All study samples were examined in duplicate with average of two concentrations used as the final value. Two quality control samples were contained in each plate analyzed in duplicate.

### Measurement of covariates

Demographic data, such as age, gender, race/ethnicity, smoking history, and medical histories were obtained from participants’ self-reported questionnaires and all data is available on the NHANES database. The participants were classified into different races such as non-Hispanic white and others. Information about medical history, including coronary artery disease, angina, hypertension, and diabetes mellitus were also collected from the participants. Waist circumference was measured in the horizontal plane between the iliac crest and the lowest rib. Laboratory data such as serum creatinine and albumin were analyzed by standard procedures.

### Statistical analysis

Statistical estimates were calculated by the Statistical Package for the Social Sciences, version 22.0 (SPSS Inc., Chicago, IL, USA) for Windows. Continuous variables were presented as means and standard deviation, while categorical variables were presented as frequencies and percentage. The differences between the gender groups in terms of demographic information and laboratory data were determined by Pearson chi-square test. A p-value ≤ 0.05 was considered statistically significant. The association between different degree of sarcopenia and higher FRS was analyzed using Logistic regression with an unadjusted and adjusted model for all pertinent variables in the study. Power statistic calculation of FRS was used to estimate the sample size in men and women.

## Results

### Characteristics of study population

The characteristic information of participants included 2597 eligible participants in this cross-sectional study was demonstrated in Table 1. The mean age was 57.60 ± 10.85 years. The mean level of serum klotho was 861.01 (pg/mL). The mean level of PAHs included 1-naphthol, 2-naphthol, 3-fluorene, 2-fluorene, 3-phenanthrene, 1-phenanthrene, 2-phenanthrene, and 1-pyrene were 43.72, 0.78, 0.29, 0.63, 0.14, 0.21, 0.11, and 0.19 (ug/L), respectively.

**Table 1:** Characteristics of Study Population

Variables	Participants (*N* = 2597)
Continuous variables, mean (SD)	
Age (years)	57.60 (10.85)
Waist circumference (cm)	101.56 (15.39)
Creatinine (mg/dL)	0.90 (0.33)
Albumin (mg/dL)	4.23 (0.30)
Klotho (pg/mL)	861.01 (308.09)
1-naphthol (ug/L)	4.37 (51.98)
2-naphthol (ug/L)	7.85 (11.29)
3-fluorene (ug/L)	0.29 (0.59)
2-fluorene (ug/L)	0.63 (1.18)
3-phenanthrene (ug/L)	0.14 (0.29)
1-phenanthrene (ug/L)	0.21 (0.33)
2-phenanthrene (ug/L)	0.11 (0.17)
1-pyrene (ug/L)	0.19 (0.39)
Category Variables, (%)	
Non-Hispanic White	1194 (46.0)
Gender (male)	1318 (50.8)
Coronary heart disease	148 (5.7)
Angina/ angina pectoris	81 (3.1)
Hypertension	647 (23.9)
Diabetes mellitus	408 (15.7)
Smoking	1299 (50.0)

### Association between PAHs and klotho

Table 2 displayed the association between PAHs and klotho. After adjusting pertinent variables included age, race, waist circumference, creatinine, albumin, medical history, and smoking history, 2-napthol and 3-fluorene were significantly associated with decreased klotho with β values -0.001 (95% confidence interval [CI]: -0.002, 0.000) and -0.026 (95%CI: -0.046, -0.005).

**Table 2:** Association between PAHs and klotho

**Variables**	**Model 1**^a^ **β**^**b**^** (95% CI)**	***P*** **Value**	**Model 2**^a^ **β**^**b**^** (95% CI)**	***P*** **Value**
	**Klotho**			
**1-napthol**	0.000 (0.000, 0.000)	0.766	0.000 (0.000, 0.000)	0.612
**2-napthol**	-0.001 (-0.002, 0.000)	0.032	-0.001 (-0.002, 0.000)	0.044
**3-fluorene**	-0.023 (-0.042, -0.003)	0.021	-0.026 (-0.046, -0.005)	0.015
**2-fluorene**	-0.010 (-0.019, 0.000)	0.057	-0.010 (-0.020, 0.000)	0.071
**3-phenanthrene**	-0.029 (-0.069, 0.011)	0.159	-0.027 (-0.067, 0.013)	0.185
**1-phenanthrene**	-0.021 (-0.057, 0.015)	0.249	-0.022 (-0.058, 0.014)	0.227
**2-phenanthrene**	-0.047 (-0.116, 0.022)	0.183	-0.044 (-0.113, 0.026)	0.219
**1-pyrene**	0.009 (-0.021, 0.038)	0.562	0.007 (-0.023, 0.037)	0.656

^a^Adjusted covariates

Model 1: unadjusted

Model 2: age, race/ethnicity, congestive heart failure, coronary heart disease, angina, smoking, hypertension, diabetes mellitus, creatinine, waist circumference, albumin

### Association between PAHs and klotho in gender difference

The relationship between PAHs and klotho in men and women was demonstrated in Table 3. After fully adjusting pertinent variables, 2-napthol, 3-fluorene, 2-fluorene, and 1-phenanthrene were significantly associated with decreased klotho in men with β values -0.002 (95%CI: -0.003, 0.000), -0.034 (95%CI: -0.059, -0.008), -0.014 (95%CI: -0.027, 0.000), and -0.049 (95%CI: -0.095, -0.004). However, no significant finding between PAHs and kloth was found in women.

**Table 3:** Association between PAHs and klotho in gender difference

	**Variables**	**Model 1**^a^ **β**^**b**^** (95% CI)**	***P*** **Value**	**Model 2**^a^ **β**^**b**^** (95% CI)**	***P*** **Value**
		**Klotho**			
**Male**	**1-napthol**	0.000 (0.000, 0.000)	0.497	0.000 (0.000, 0.000)	0.379
	**2-napthol**	-0.002 (-0.003, 0.000)	0.029	-0.002 (-0.003, 0.000)	0.038
	**3-fluorene**	-0.028 (-0.052, -0.004)	0.023	-0.034 (-0.059, -0.008)	0.009
	**2-fluorene**	-0.012 (-0.025, 0.000)	0.056	-0.014 (-0.027, 0.000)	0.046
	**3-phenanthrene**	-0.036 (-0.082, 0.010)	0.129	-0.040 (-0.086, 0.007)	0.093
	**1-phenanthrene**	-0.046 (-0.091, 0.000)	0.049	-0.049 (-0.095, -0.004)	0.035
	**2-phenanthrene**	-0.070 (-0.157, 0.016)	0.111	-0.078 (-0.165, 0.009)	0.078
	**1-pyrene**	0.014 (-0.020, 0.048)	0.410	0.011 (-0.023, 0.046)	0.513
**Female**	**1-napthol**	0.000 (0.000, 0.000)	0.701	0.000 (0.000, 0.000)	0.815
	**2-napthol**	-0.001 (-0.002, 0.001)	0.284	-0.001 (-0.002, 0.001)	0.336
	**3-fluorene**	-0.014 (-0.045, 0.017)	0.381	-0.015 (-0.049, 0.018)	0.376
	**2-fluorene**	-0.006 (-0.021, 0.009)	0.461	-0.005 (-0.021, 0.011)	0.507
	**3-phenanthrene**	-0.005 (-0.076, 0.065)	0.881	-0.011 (-0.083, 0.060)	0.756
	**1-phenanthrene**	0.006 (-0.049, 0.061)	0.839	0.004 (-0.052, 0.059)	0.899
	**2-phenanthrene**	-0.001 (-0.109, 0.107)	0.985	-0.009 (-0.120, 0.102)	0.870
	**1-pyrene**	0.006 (-0.046, 0.058)	0.824	0.000 (-0.054, 0.054)	0.997

^a^Adjusted covariates:

Model 1: unadjusted

Model 2: age, race/ethnicity, congestive heart failure, coronary heart disease, angina, smoking, hypertension, diabetes mellitus, creatinine, waist circumference, albumin

## Discussion

In the cross-sectional study, we investigated the relationship between exposure to PAHs and serum klotho levels in a nationally representative sample among American population. PAHs such as 2-napthol and 3-fluorene were significantly associated with decreased klotho levels. Especially, this relationship remained significant in men, including 2-napthol, 3-fluorene, 2-fluorene, and 1-phenanthrene, respectively. To the best of our knowledge, no previous study has reported the association between PAH exposure and serum klotho. Our study was the first to investigate the plausible pathway of PAHs exposure involving the aging process.

Klotho gene is reported contributing to anti-aging property by telomerase activation and consequently telomere maintenance [35]. Ullah et al. demonstrated that klotho protein deficiency diminished telomerase activity by impairing the expression of telomeric repeat-binding factor 1, and then led to altered differentiation, cellular senescence, and stem cell apoptosis [36]. Numerous evidence has reported the association between PAH exposure and telomere length. Adli et al. displayed an inverse association between urinary 1-hydroxipayrene level and telomere length in preschool children [37]. An emerging study suggested that everyday life exposure to PAH reduces leukocyte telomere length and mitochondrial DNA copy number [38]. Overall, these studies might support our findings that PAH exposure was associated with reduced serum klotho levels.

Aging is a complicated phenomenon and is influenced by various genetic and environmental factors [39]. Cigarette smoking is regarded as one of the important risk factors of aging process [40]. In a retrospective study included Japanese adults, smokers showed significantly higher serum levels of klotho protein than non-smokers [41]. Kamizono et al. reported cigarette smoking was significantly associated with plasma α-klotho levels [42]. As one of the possible or known human carcinogens, PAHs are kinds of complex chemical composition released by cigarette smoking [43], and potentially affecting the aging process. Best et al. demonstrated the adverse effect of PAHs exposure on cognitive function among elderly population [44]. Another study also reported exposure to PAHs correlated with functional dependence among older people in the United States [39]. Recently, a variety of evidence suggested that the states of DNA methylation play an important role in gene regulation associated with aging process [45]. In a vitro study, Duca et al. demonstrated that PAH exposure affected DNA and RNA methylation in a rat model [46]. Li et al. reported that PAH exposure was associated with acceleration of DNA methylation in a population-based study [32]. Hypermethylation of klotho promoter decreases the expression of klotho protein in the progress of chronic kidney disease [47]. Genetic silencing of klotho in young muscle progenitor cell induce mitochondrial DNA damage and decreased cellular bioenergetics leading to aging process of muscle regeneration [48].

PAHs are well-known endocrine disrupting toxicants and reproductive chemicals [49]. Zhang et al. demonstrates that high-molecular weight PAHs can activate their endocrine-disrupting effects by androgen or estrogen receptors, with structure-dependent agonistic or antagonistic effects [50]. Drwal et al. provides evidence of cell specific effects of low-molecular weight PAHs on proliferation, the cell cycle, and hormone secretion [51]. Highly prevalent low-molecular weight PAHs are recognized as inhibitors of gap junctional intercellular communication (GJIC) in some types of cells [52]. GJIC is suggested a key driver of endocrine function in testis, which makes testicular GJIC as a target for PAHs [53]. In a recent study, PAHs are considered an important risk factor leading to reproductive dysfunctions in men through impaired testicular GJIC [54]. Accordingly, the sex-specific impact of PAHs may elucidate the significant change with serum klotho in men of our study.

There are some limitations should be concerned when interpreting our results. First, the cross-sectional design of this study prevented the identification of causal nature between PAHs exposure and serum klotho. PAH metabolite biomarkers display recent exposures rather than long-term exposures. Second, this study assessed serum klotho levels only over the short term. The long-term effect of PAH exposure on klotho will need to be elucidated in the future. Last, the mechanisms of PAH exposure on serum klotho levels are still unknown. Further long-term examination are needed to provide more evidence of air pollution control and prevention of aging acceleration.

## Conclusion

In the present study, we demonsrtrated that PAH exposure was associated with decreased serum klotho levels, highlighting the negative impact of PAH exposure on aging. Future studies are warranted to investigate the potential mechanisms of klotho gene expression and clinical significance influenced by such environment-related effects.

## Data Availability

The datasets generated and analyses performed during the current study are not publicly available due to the consent requirement of participants, but sex and age decade-stratified descriptive data are available from the corresponding author on reasonable request.

## References

[1] Shimada T, Kakitani M, Yamazaki Y, Hasegawa H, Takeuchi Y, Fujita T, Fukumoto S, Tomizuka K, Yamashita T (2004). Targeted ablation of Fgf23 demonstrates an essential physiological role of FGF23 in phosphate and vitamin D metabolism. J Clin Investig.

[2] Vervloet MG, Adema AY, Larsson TE, Massy ZA (2014). The role of klotho on vascular calcification and endothelial function in chronic kidney disease. Semin Nephrol.

[3] Zhou X, Yang Q, Xie Y, Sun J, Hu J, Qiu P, Cao W, Wang S (2015). Tetrahydroxystilbene glucoside extends mouse life span via upregulating neural klotho and downregulating neural insulin or insulin-like growth factor 1. Neurobiol Aging.

[4] Hu MC, Shiizaki K, Kuro-o M, Moe OW (2013). Fibroblast growth factor 23 and Klotho: physiology and pathophysiology of an endocrine network of mineral metabolism. Annu Rev Physiol.

[5] Kuro OM (2019). The Klotho proteins in health and disease. Nat Rev Nephrol.

[6] Kim HJ, Lee J, Chae D-W, Lee K-B, Sung SA, Yoo T-H, Han SH, Ahn C, Oh K-H (2019). Serum klotho is inversely associated with metabolic syndrome in chronic kidney disease: results from the KNOW-CKD study. BMC Nephrol.

[7] Zou D, Wu W, He Y, Ma S, Gao J (2018). The role of klotho in chronic kidney disease. BMC Nephrol.

[8] Semba RD, Cappola AR, Sun K, Bandinelli S, Dalal M, Crasto C, Guralnik JM, Ferrucci L (2011). Plasma klotho and cardiovascular disease in adults. J Am Geriatr Soc.

[9] Fetzer JC (2007). THE CHEMISTRY AND ANALYSIS OF LARGE PAHs. Polycyclic Aromat Compd.

[10] Bocchi C, Bazzini C, Fontana F, Pinto G, Cassoni F (2017). Genotoxicity of airborne PM(2 5) assessed by salmonella and comet assays in five cities of the Emilia-Romagna (Italy) mutagenicity monitoring network. Environmental and molecular mutagenesis.

[11] Idowu O, Semple KT, Ramadass K, O'Connor W, Hansbro P, Thavamani P (2019). Beyond the obvious: Environmental health implications of polar polycyclic aromatic hydrocarbons. Environ Int.

[12] ChooChuay C, Pongpiachan S, Tipmanee D, Suttinun O, Deelaman W, Wang Q, Xing L, Li G, Han Y, Palakun J (2020). Impacts of PM2 5 sources on variations in particulate chemical compounds in ambient air of Bangkok Thailand. Atmospheric Pollution Research.

[13] Deelaman W, Pongpiachan S, Tipmanee D, Suttinun O, Choochuay C, Iadtem N, Charoenkalunyuta T, Promdee K (2020). Source apportionment of polycyclic aromatic hydrocarbons in the terrestrial soils of King George Island Antarctica. Journal of South American Earth Sciences.

[14] LundeHermansson A, Hassellöv I-M, Moldanová J, Ytreberg E (2021). Comparing emissions of polyaromatic hydrocarbons and metals from marine fuels and scrubbers. Transportation Research Part D Transport and Environment.

[15] Abdel-Shafy HI, Mansour MSM (2016). A review on polycyclic aromatic hydrocarbons: Source, environmental impact, effect on human health and remediation. Egypt J Pet.

[16] Låg M, Øvrevik J, Refsnes M, Holme JA (2020). Potential role of polycyclic aromatic hydrocarbons in air pollution-induced non-malignant respiratory diseases. Respir Res.

[17] Deelaman W, Pongpiachan S, Tipmanee D, Choochuay C, Suttinun O, Charoenkalunyuta T, Promdee K (2021). Ecotoxicological risk and health risk characterization of polycyclic aromatic hydrocarbons (PAHs) in terrestrial soils of King George Island. Antarctica. Polar Science.

[18] White AJ, Bradshaw PT, Herring AH, Teitelbaum SL, Beyea J, Stellman SD, Steck SE, Mordukhovich I, Eng SM, Engel LS (2016). Exposure to multiple sources of polycyclic aromatic hydrocarbons and breast cancer incidence. Environ Int.

[19] Zhang Y, Tao S, Shen H, Ma J (2009). Inhalation exposure to ambient polycyclic aromatic hydrocarbons and lung cancer risk of Chinese population. Proc Natl Acad Sci USA.

[20] Zhou Y, Sun H, Xie J, Song Y, Liu Y, Huang X, Zhou T, Rong Y, Wu T, Yuan J (2016). Urinary Polycyclic Aromatic Hydrocarbon Metabolites and Altered Lung Function in Wuhan, China. Am J Respir Crit Care Med.

[21] Li Z, Romanoff LC, Lewin MD, Porter EN, Trinidad DA, Needham LL, Patterson DG, Sjödin A (2010). Variability of urinary concentrations of polycyclic aromatic hydrocarbon metabolite in general population and comparison of spot, first-morning, and 24-h void sampling. J Eposure Sci Environ Epidemiol.

[22] Pratt MM, John K, MacLean AB, Afework S, Phillips DH, Poirier MC (2011). Polycyclic aromatic hydrocarbon (PAH) exposure and DNA adduct semi-quantitation in archived human tissues. Int J Environ Res Public Health.

[23] Patel AP, Mehta SS, White AJ, Niehoff NM, Arroyave WD, Wang A, Lunn RM (2021). Urinary polycyclic aromatic hydrocarbon metabolites and mortality in the United States A prospective analysis. PloS one.

[24] Alhamdow A, Lindh C, Albin M, Gustavsson P, Tinnerberg H, Broberg K (2019). Cardiovascular disease-related serum proteins in workers occupationally exposed to polycyclic aromatic hydrocarbons. Toxicological sciences : an official journal of the Society of Toxicology.

[25] Alhamdow A, Tinnerberg H, Lindh C, Albin M, Broberg K (2019). Cancer-related proteins in serum are altered in workers occupationally exposed to polycyclic aromatic hydrocarbons: a cross-sectional study. Carcinogenesis.

[26] Castaño-Vinyals G, D'Errico A, Malats N, Kogevinas M (2004). Biomarkers of exposure to polycyclic aromatic hydrocarbons from environmental air pollution. Occupational and environmental medicine.

[27] Moorthy B, Chu C, Carlin DJ (2015). Polycyclic aromatic hydrocarbons: from metabolism to lung cancer. Toxicol Sci.

[28] Rengarajan T, Rajendran P, Nandakumar N, Lokeshkumar B, Rajendran P, Nishigaki I (2015). Exposure to polycyclic aromatic hydrocarbons with special focus on cancer. Asian Pac J Trop Biomed.

[29] Holme JA, Brinchmann BC, Refsnes M, Låg M, Øvrevik J (2019). Potential role of polycyclic aromatic hydrocarbons as mediators of cardiovascular effects from combustion particles. Environ Health.

[30] Chen YY, Kao TW, Wang CC, Wu CJ, Zhou YC, Chen WL (2020). Association between polycyclic aromatic hydrocarbons exposure and bone turnover in adults. Eur J Endocrinol.

[31] Chen YY, Wang CC, Kao TW, Yang HF, Sun YS, Chen WL (2020). Detrimental association between quadriceps strength and exposure to polycyclic aromatic hydrocarbons in elderly adults Applied physiology, nutrition, and metabolism. Physiologie appliquee nutrition et metabolism.

[32] Li J, Zhu X, Yu K, Jiang H, Zhang Y, Wang B, Liu X, Deng S, Hu J, Deng Q (2018). Exposure to Polycyclic Aromatic Hydrocarbons and Accelerated DNA Methylation Aging. Environ Health Perspect.

[33] Wang Y, Meng L, Pittman EN, Etheredge A, Hubbard K, Trinidad DA, Kato K, Ye X, Calafat AM (2017). Quantification of urinary mono-hydroxylated metabolites of polycyclic aromatic hydrocarbons by on-line solid phase extraction-high performance liquid chromatography-tandem mass spectrometry. Anal Bioanal Chem.

[34] (CDC) CfDCaP: National Health and Examination Survey. Data Documentation, Codebook, and Frequencies. Phthalates, Phytoestrogens, & PAHs–Urine 2012.

[35] Yamamoto M, Clark JD, Pastor JV, Gurnani P, Nandi A, Kurosu H, Miyoshi M, Ogawa Y, Castrillon DH, Rosenblatt KP (2005). Regulation of oxidative stress by the anti-aging hormone klotho. J Biol Chem.

[36] Ullah M, Sun Z (2019). Klotho Deficiency Accelerates Stem Cells Aging by Impairing Telomerase Activity. J Gerontol A Biol Sci Med Sci.

[37] Adli A, Hosseini SM, LariNajafi M, Behmanesh M, Ghezi E, Rasti M, Kazemi AA, Rad A, Falanji F, Mohammadzadeh M (2021). Polycyclic aromatic hydrocarbons exposures and telomere length A cross-sectional study on preschool children. Environmental Research.

[38] Pavanello S, Campisi M, Mastrangelo G, Hoxha M, Bollati V (2020). The effects of everyday-life exposure to polycyclic aromatic hydrocarbons on biological age indicators. Environ Health.

[39] Chen YY, Kao TW, Wang CC, Chen YJ, Wu CJ, Lai CH, Chen WL (2019). Exposure to polycyclic aromatic hydrocarbons and risk of disability among an elderly population. Environ Sci Pollut Res Int.

[40] Nicita-Mauro V, Basile G, Maltese G, Nicita-Mauro C, Gangemi S, Caruso C (2008). Smoking, health and ageing. Immun Ageing.

[41] Nakanishi K, Nishida M, Harada M, Ohama T, Kawada N, Murakami M, Moriyama T, Yamauchi-Takihara K (2015). Klotho-related Molecules Upregulated by Smoking Habit in Apparently Healthy Men: A Cross-sectional Study. Sci Rep.

[42] Kamizono Y, Shiga Y, Suematsu Y, Imaizumi S, Tsukahara H, Noda K, Kuwano T, Fujimi K, Saku K, Miura SI (2018). Impact of cigarette smoking cessation on plasma α-klotho levels. Medicine.

[43] Vu AT, Taylor KM, Holman MR, Ding YS, Hearn B, Watson CH (2015). Polycyclic Aromatic Hydrocarbons in the Mainstream Smoke of Popular U S Cigarettes. Chem Res Toxicol.

[44] Best EA, Juarez-Colunga E, James K, LeBlanc WG, Serdar B (2016). Biomarkers of Exposure to Polycyclic Aromatic Hydrocarbons and Cognitive Function among Elderly in the United States (National Health and Nutrition Examination Survey 2001–2002). PloS one.

[45] Jones PA (2012). Functions of DNA methylation: islands, start sites, gene bodies and beyond. Nat Rev Genet.

[46] Duca R-C, Grova N, Ghosh M, Do J-M, Hoet PHM, Vanoirbeek JAJ, Appenzeller BMR, Godderis L (2018). Exposure to Polycyclic Aromatic Hydrocarbons Leads to Non-monotonic Modulation of DNA and RNA (hydroxy)methylation in a Rat Model. Sci Rep.

[47] Chen J, Zhang X, Zhang H, Lin J, Zhang C, Wu Q, Ding X (2013). Elevated Klotho promoter methylation is associated with severity of chronic kidney disease. PLoS ONE.

[48] Sahu A, Mamiya H, Shinde SN, Cheikhi A, Winter LL, Vo NV, Stolz D, Roginskaya V, Tang WY, St. Croix C (2018). Age-related declines in α-Klotho drive progenitor cell mitochondrial dysfunction and impaired muscle regeneration. Nature Communications.

[49] Ramesh A, Harris KJ, Archibong AE (2017). Reproductive toxicity of polycyclic aromatic hydrocarbons.

[50] Zhang Y, Dong S, Wang H, Tao S, Kiyama R (2016). Biological impact of environmental polycyclic aromatic hydrocarbons (ePAHs) as endocrine disruptors. Environmental pollution (Barking Essex 1987).

[51] Drwal E, Rak A, Grochowalski A, Milewicz T, Gregoraszczuk EL (2017). Cell-specific and dose-dependent effects of PAHs on proliferation, cell cycle, and apoptosis protein expression and hormone secretion by placental cell lines. Toxicol Lett.

[52] Osgood RS, Upham BL, Hill III T, Helms KL, Velmurugan K, Babica P, Bauer AK (2013). Polycyclic Aromatic Hydrocarbon-Induced Signaling Events Relevant to Inflammation and Tumorigenesis in Lung Cells Are Dependent on Molecular Structure. PloS one.

[53] Meda P (2018). Gap junction proteins are key drivers of endocrine function. Biochimica et Biophysica Acta (BBA) Biomembranes.

[54] Kubincová P, Sychrová E, Raška J, Basu A, Yawer A, Dydowiczová A, Babica P, Sovadinová I (2019). Polycyclic Aromatic Hydrocarbons and Endocrine Disruption: Role of Testicular Gap Junctional Intercellular Communication and Connexins. Toxicological sciences : an official journal of the Society of Toxicology.

